# Causal association of gut microbiota and esophageal cancer: a Mendelian randomization study

**DOI:** 10.3389/fmicb.2023.1286598

**Published:** 2023-12-01

**Authors:** Xiangyu Gao, Zhiguo Wang, Bowen Liu, Yufeng Cheng

**Affiliations:** ^1^Department of Radiation Oncology, Qilu Hospital of Shandong University, Jinan, China; ^2^The Second Hospital, Cheeloo College of Medicine, Shandong University, Jinan, China

**Keywords:** causality, gut microbiota, Mendelian randomization, esophageal cancer, SNPs

## Abstract

**Introduction:**

Despite the growing body of evidence, the link between the gut microbiota and different types of tumors, such as colorectal, gastric, and liver cancer, is becoming more apparent. The gut microbiota can be used as a reference for evaluating various diseases, including cancer, and can also act as risk factors or preventive factors. However, the specific connection between the gut microbiota and the advancement of esophageal cancer has yet to be investigated. Therefore, the aim of this research is to clarify the possible causal influence of intestinal microorganisms on the vulnerability to esophageal cancer through the utilization of Mendelian randomization (MR) studies.

**Methods:**

In this study, we employed a two-sample Mendelian randomization approach to evaluate the unbiased causal association between 150 different gut microbiota types and the occurrence of esophageal cancer. Following the selection from the IEU GWAS database and SNP filtration, we utilized various MR statistical techniques on the suitable instrumental variables. These included IVW methods, employing inverse variance weighting. Additionally, we performed a range of sensitivity analyses to confirm the heterogeneity and pleiotropy of the instrumental variables, thus ensuring the reliability of the outcomes.

**Results:**

The increased likelihood of developing esophageal cancer is linked to the genetically predicted high levels of *Gordonibacter, Oxalobacter, Coprobacter, Veillonella, Ruminiclostridium 5, Ruminococcus 1*, and *Senegalimasilia genera*. Conversely, a decreased risk of esophageal cancer is associated with the high abundance of *Turicibacter, Eubacterium oxidoreducens group, Romboutsia*, and *Prevotella 9 genera*. No heterogeneity and pleiotropy were detected in the sensitivity analysis.

**Discussion:**

We found that 11 types of gut microbial communities are associated with esophageal cancer, thereby confirming that the gut microbiota plays a significant role in the path.

## Introduction

In the past few decades, there has been a gradual rise in the occurrence of esophageal cancer and other ailments affecting the esophagus. This poses a notable danger to human well-being and has a direct impact on the overall quality of life for individuals. Esophageal cancer is widely acknowledged as one of the prevalent tumors affecting the digestive tract globally, with its occurrence and fatality rate being among the highest 10. Approximately, 300,000 deaths occur from esophageal cancer each year globally. Although the survival rate of esophageal cancer has increased in recent decades and patients who have early-stage cancer and undergo radical surgery have a relatively positive prognosis, the initial signs of esophageal cancer are frequently inconspicuous and can be easily disregarded. The majority of individuals will consult a healthcare professional once they encounter worsening difficulty swallowing and discomfort in the chest, both of which are evident indications. Unfortunately, at this point, they are already in the advanced or late phase of esophageal cancer, leading to missed chances for surgery and the ideal treatment timeframe. This proportion of patients in such conditions can reach 90%. As a result, individuals diagnosed with esophageal cancer experience a diminished quality of life, elevated mortality rate, and unfavorable prognosis, as indicated by a less than 20% survival rate over a 5-year period (Zhang et al., 2023), leading to a substantial burden of illness. Hence, it is imperative to possess a thorough comprehension of the origin of esophageal cancer and enforce preventive measures at the initial stage to decrease its occurrence.

Over the past few years, an increasing amount of research has emphasized the complex interaction between the microbiome of the human digestive system and cancer, emphasizing the possible influence of the gut microbiome on the start, advancement, and reaction to therapy of cancer. The gastrointestinal tract serves as an extension of the human body’s natural environment, offering an ideal habitat and abundant nutrients for the microbiota. Among the various body sites, the human gut microbiota stands out as a prominent component of the human microbiome, displaying the highest bacterial abundance and diversity. Although our knowledge of the esophageal microbiome is still restricted, it is widely recognized that the esophagus, located between the oropharynx and the stomach, is not a sterile part of the digestive system and is inhabited by various microorganisms. Boasting a substantial mucosal surface area, the esophagus harbors a rich and heterogeneous microbial community. Microorganisms can readily access the esophagus through swallowing and reflux events. The gut microbiome, which extends throughout the digestive system, is acknowledged as a potential ecological element that impacts human well-being (Long et al., 2023). Recent findings have shown a significant association between the gut microbiome and different types of tumors, such as colorectal, gastric, and hepatic cancers (Eun et al., 2014; Gopalakrishnan et al., 2018; Ma et al., 2018; Routy et al., 2018; Lv et al., 2019a). The gut microbiome has become a valuable point of reference in the diagnosis, treatment, and prognosis of various illnesses, such as cancer, while also playing a role as a risk factor or preventive measure. Should the established correlation between gut microbiota traits and cancer risk be confirmed as having a causal foundation, the gut microbiota may emerge as a hopeful focus for the early detection and prevention of cancer (Wei et al., 2023).

At present, there is a scarcity of research on the gut microbiota attributes among individuals with esophageal cancer, with the majority of investigations being observational in nature. Nonetheless, conventional observational research encounters difficulties in establishing a causal relationship between gut microbiota and the risk of cancer, given their vulnerability to confounding variables like eating patterns, age, and the impact of reverse causation. Hence, it is imperative to have a dependable approach for investigating the cause-and-effect connection.

Over the past 10 years, extensive genome-wide association studies (GWAS) have transformed the realm of complex disease genetics. These studies have analyzed countless genetic variations in numerous individuals to reveal connections between genotypes and phenotypes (Visscher et al., 2017). Significantly advancing our understanding, the domain of oncology has notably identified more than 450 genetic variations associated with an increased susceptibility to common cancers (Long et al., 2023). The application of GWAS information in clinical settings has created new possibilities for cancer prevention, presenting valuable prospects in the domain (Sud et al., 2017).

Mendelian randomization (MR) is an instrumental variable method that employs genetic variation in human populations as instrumental variables to mimic clinical traits. MR utilizes instrumental variable analysis to mimic the randomization process of causality inference in randomized controlled trials (RCTs) in order to mitigate biases found in conventional epidemiological studies. Because genetic variation occurs randomly and is determined at conception (You et al., 2021), the findings from MR analysis are highly unaffected by confounding factors and reverse causality commonly found in traditional observational studies. This enhances their persuasiveness and reliability. The use of MR has been extensive in investigating possible causal connections between environmental exposures and diseases. For this research, we performed a two-sample MR analysis to establish a causal link between the microbiota of the human gut and its metabolites with regard to esophageal cancer. Our goal was to reveal the potential influence of gut microbiota on the likelihood of developing esophageal cancer and establish a theoretical foundation for the early detection and treatment of this disease.

## Materials and methods

### Selection of datasets

For MR analysis, we chose exposure datasets and outcome datasets from the MRCIEU database. This database includes a vast compilation of 227,808,842,007 genetic associations derived from 40,027 GWAS summary datasets, which can be accessed for querying or downloading.

To examine the unbiased causal connection between the occurrence of esophageal cancer and the human gut microbiota, a two-sample MR method was utilized in this research. The abundance of different human gut microbiota served as the exposure factor, while the occurrence of esophageal cancer was considered as the outcome factor. One of the largest meta-analyses of microbial GWAS (mGWAS) on bacterial abundance, which included 150 human gut microbiota GWAS datasets, resulted in the incorporation of 32 phyla and 118 genera (Kurilshikov et al., 2021). According to Kurilshikov’s research, Supplementary Table 1 shows a description of the participants in each cohort in a dataset of gut microbiota. Data related to esophageal cancer were obtained from the public database IEU Open GWAS (MR Base; https://gwas.mrcieu.ac.uk/). The GWAS summary dataset ID “ieu-b-4960” was chosen to analyze the genetic variations linked to esophageal cancer. The data for this genome-wide association study (GWAS) were obtained from the United Kingdom Biobank, with the most recent update in 2021. It consisted of 740 individuals diagnosed with esophageal cancer and 372,016 individuals serving as controls. MR studies often encounter bias due to population stratification, as the frequencies of alleles for the same single nucleotide polymorphism (SNP) can differ across ancestral populations. In order to reduce the influence of population stratification, we only included samples of European origin in our study. These samples consisted of 372,756 individuals of European descent, providing a total of 8,970,465 SNPs for analysis (Emdin et al., 2017). Supplementary Table 2 shows information about datasets. Ethical approval was not necessary for our analysis as it relied on published studies or publicly available GWAS summary data, without involving the collection of original data.

### Study design

**Figure 1:**
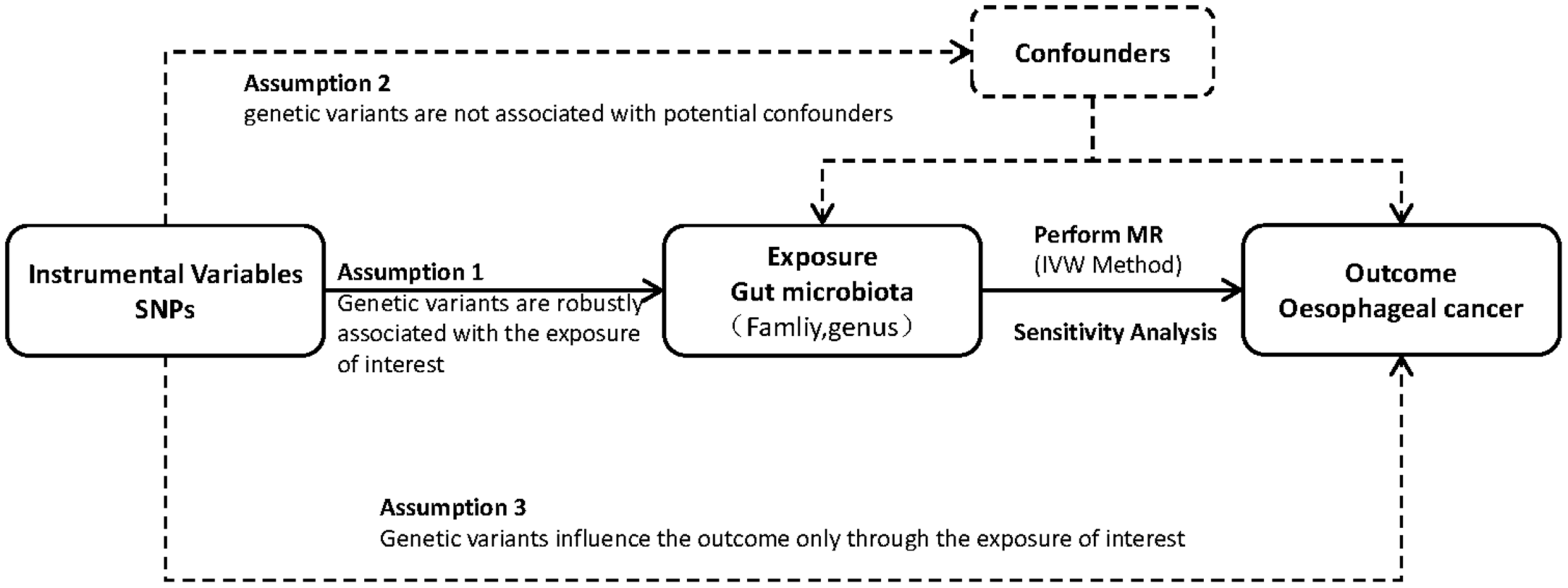
Study design. An overview of the study design.

To explore the link between the risk of esophageal cancer and gut microbiota, we utilized a two-sample MR method. Figure 1 visually illustrates the study design. This study utilized single nucleotide polymorphisms (SNPs) as instrumental factors to assess the correlation between the exposure element (microbial composition of the human gastrointestinal tract) and the outcome element (esophageal carcinoma). The successful implementation of MR analysis depends on three crucial assumptions: pertinence, which implies that genetic variations are strongly linked to the exposure factor; autonomy, which means that genetic variations are not influenced by any confounding factors that may affect the relationship between exposure and outcome; and exclusiveness, which suggests that genetic variations only affect the outcome through the exposure factor. In order to clarify these presumptions, we created a directed acyclic graph (DAG) that includes the instrumental variable (SNP), the factor that influences the exposure (human gut microbiota), and the result (esophageal cancer).

### Selection of tool variables

We conducted a series of quality control measures on the acquired GWAS summary data to choose suitable SNPs, in accordance with the underlying principles of MR.

Initially, we chose SNPs that exhibited noteworthy association with the exposure factor by employing a parameter threshold of genome-wide importance (*p* < 1 × 10^−5^). In order to guarantee the independence and genetic uniqueness of the instrumental variables, we chose SNPs with an *r*^2^ < 0.001 and physical distance > 10,000 kb in regions of no linkage disequilibrium (LD). Considering the potential missing SNP data in the outcome database, we substituted the target SNPs with high LD (*r*^2^ > 0.8) proxy SNPs when they were not available in the study results. Ambiguous SNPs in the matching process and palindrome SNPs with intermediate allele frequencies (allele frequency between 0.01 and 0.30 and SNPs with A/T or G/C alleles) were excluded from the selected SNPs in this study. In addition, we computed the *F*-value for SNPs to assess their efficacy as instrumental variables (IVs). The *F*-statistic evaluates the effectiveness of the instrumental variables in elucidating the risk factor and is calculated using the equation *F* = *b*^2^/*se*^2^. IVs with an *F*-statistic <10 were considered weak instruments and excluded to ensure strong correlation between SNPs and the exposure factor (Burgess et al., 2017). To investigate the causal estimation direction of each SNP, we utilized the MR-Steiger test and eliminated SNPs with inaccurate directions (Li et al., 2022). Phenoscanner was utilized to examine the potential connections between every SNP and confounding variables, while excluding SNPs that could potentially violate the assumption of independence by being linked to potential confounders (Staley et al., 2016). To test and eliminate outlier SNPs, MR-PRESSO analysis was ultimately conducted.

Following the previously mentioned thorough screening process, the chosen SNPs will serve as the ultimate instrumental variables for MR analysis.

### Mendelian randomization analysis

We meticulously chose suitable SNPs as instrumental variables based on the mentioned selection criteria and utilized a rigorous two-sample MR approach to examine the potential causal influence of the exposure factor (human gut microbiota) on the vulnerability to the outcome (esophageal cancer). In order to thoroughly assess the findings and tackle possible biases, we employed various MR statistical approaches, such as the IVW method which utilizes inverse variance-weighting, MR-Egger regression, the WM method which applies weighting based on medians, the weighted mode method, and the MR-RAPS method. To ensure the robustness and reliability of our analysis, we utilized these approaches to evaluate variability among the estimates and investigate the existence of pleiotropic effects.

To obtain the most precise estimation, the inverse variance-weighted (IVW) technique, which is widely acknowledged as the leading approach in MR analysis and the primary method used in this research, was employed. The IVW approach combines the ratio estimates acquired for each SNP, yielding an estimation of the impact of the exposure factor on the outcome (Burgess et al., 2013). The Wald ratio estimate measures the ratio of the effect of a single SNP on the outcome to its effect on the risk factor, under the assumption that all associations adhere to a log-linear relationship (Thomas and Conti, 2004).

We utilized MR-Egger regression, a valuable tool in MR analysis, to establish a weighted linear regression between the coefficients of the outcome and exposure. MR-Egger regression, like the IVW method, can handle the existence of horizontal pleiotropy, and the intercept term in MR-Egger can evaluate the magnitude of horizontal pleiotropy. This approach allows for one or multiple genetic variants to exhibit pleiotropic effects. When there is noticeable horizontal pleiotropy identified in the analysis findings, we prioritize the causal estimation outcomes derived from the MR-Egger approach. The MR-Egger method obtains the ratio estimate by regressing the ratio of the SNP’s correlation with the outcome and the SNP’s correlation with the exposure, using the negative variances of the outcome correlations as weights (Burgess and Thompson, 2017). It is important to mention that the NOME assumption is a key requirement for the MR-Egger method. In order to evaluate possible breaches of the NOME assumption through the MR-Egger method, we computed the *I*^2^ metric, which measures the extent of deviation from the assumption (Bowden et al., 2016). Correction is deemed necessary when the *I*^2^ value falls below 90%.

Incorporating individual genetic variants with significant outlier causal estimates, the WM method demonstrates improved resilience in comparison to the IVW and MR-Egger methods. This method proves particularly reliable in scenarios where the inclusion of such variants is necessary. The WM approach functions by computing normalized inverse-variance weights for every genetic variant and then merging these weights to produce the estimates. Importantly, the WM approach provides reliable estimates of causal effects as long as a minimum of 50% of the weights utilized in the analysis come from valid instrumental variables. The WM method can accurately estimate causal relationships, enhancing precision even when there are some invalid instrumental variables.

To investigate potential pleiotropy in the IVW model and detect any outliers, we performed MR-PRESSO analysis, which is based on Mendelian randomization and residual sum. After identifying these SNPs, we proceeded to analyze the data again using the IVW technique, making sure that the outcomes were not affected by the previously detected anomalies. Furthermore, this research utilized the newly created MR-RAPS technique, which directly models the pleiotropic impacts of genetic variations using a random-effects distribution. The novel strategy offers enhanced resilience in contrast to conventional Mendelian randomization techniques (Zhao et al., 2020). We estimated the statistical power of our study by utilizing an online power calculator (Burgess, 2014). The final outcomes were deemed statistically significant with a significance level of *p* < 0. 05.

### Sensitivity analysis

Even when all SNPs are valid instrumental variables, heterogeneity may still exist among these SNPs. The reliability of causal conclusions decreases when there is significant diversity, particularly if the evidence for causal effects is based on only one or a few SNPs that have outliers or causal effects. Initially, we computed the diversity among SNPs utilizing the IVW approach and confirmed it through the Cochran’s Q test. Heterogeneity was deemed to exist if the *p* of the Q statistic was less than 0.05. Comprehensive analysis was conducted using a multiplicative random-effects model and a weighted median model in the presence of heterogeneity. Next, we utilized the leave-one-out method (eliminating one SNP at a time from the MR analysis) to evaluate the impact of individual SNPs on the conclusions of the MR analysis. Moreover, we utilized the MR-Steiger approach to confirm the direction of causal estimates, guaranteeing the strength of the findings.

### Data visualization

In the concluding section for visualization, this research graphed scatter plots for every SNP in relation to exposure factors and outcome effects, accompanied by regression curves displaying causal estimates. Significance heatmap of MR analysis was plotted to show the result. To evaluate possible directional effects and pleiotropy, as well as examine the distribution of data, funnel plots were employed. The final causal estimates were used to create forest plots, which showed the results of each SNP and the overall MR analysis.

### Statistical software

The statistical analyses and data visualizations for Mendelian randomization were conducted using R software version 4.1.2, employing the packages “TwoSampleMR” and “MR-PRESSO”.

## Results

To investigate the causal connection between characteristics of the human gut microbiome and the risk of esophageal cancer, we performed a two-sample MR analysis utilizing summary statistics from GWAS. Following the implementation of the threshold for genome-wide significance, we obtained a grand total of 1,918 SNPs that were linked to 150 microbial traits. All instrumental variables had F-statistics exceeding 10. Because of the absence of data for certain SNPs in the outcome dataset, we eliminated 65 pertinent SNPs. We identified 294 palindromic or ambiguous SNPs associated with human gut microbiome characteristics, which will be excluded. The aforementioned findings suggest that the chosen SNPs adhere to the presumption of correlation in MR analysis. After conducting MR-Steiger tests, no SNP exhibiting an incorrect causal estimate direction was identified, thereby eliminating the need for removal. By utilizing the MR-PRESSO test and Phenoscanner test, we eliminated 19 SNPs that were linked to potential confounding variables like smoking (Doll et al., 1994; Freedman et al., 2007; Smyth et al., 2017), alcohol consumption (Yang et al., 2005; Prabhu et al., 2014), and obesity (Chow et al., 1998; Veugelers et al., 2006; Lagergren, 2011) during the pleiotropy analysis. As a result of violating the assumption of independence in MR analysis, we excluded the SNPs that were linked to confounding factors. Through the MR-PRESSO test, we discovered seven SNPs exhibiting horizontal pleiotropy. Following the application of Bonferroni adjustment, we eliminated five SNPs that were directly associated with the result. After a thorough screening process, a total of 1,528 SNPs were chosen as suitable instrumental variables for the conclusive MR analysis.

To validate horizontal pleiotropy, we computed the intercept term of MR-Egger regression after excluding SNPs associated with the exposure factor, taking into account the possibility of other biological effects of certain SNPs that might impact the outcome. There was no indication of horizontal pleiotropy.

**Figure 2:**
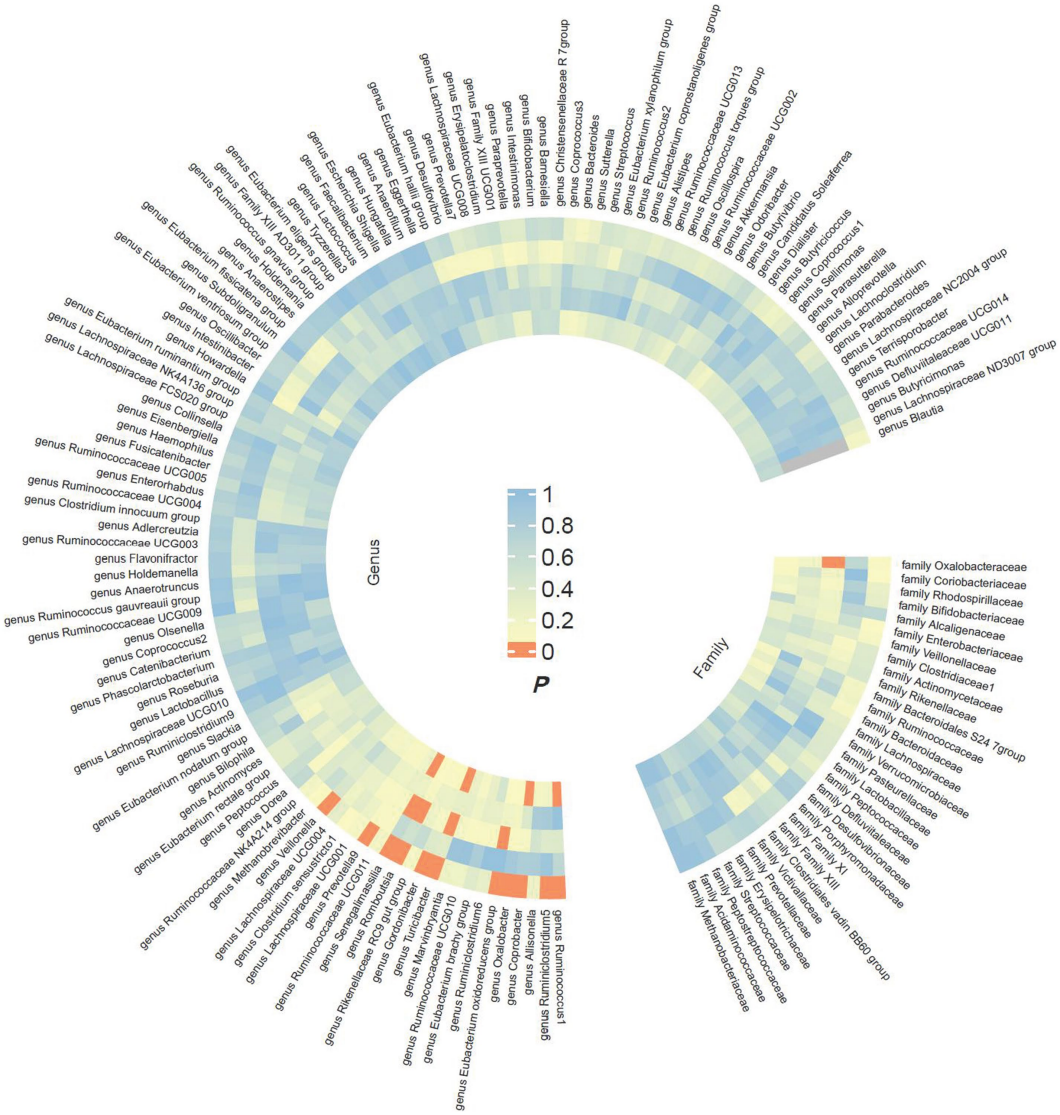
Preliminary MR estimates for the associations between gut microbiota and the risk of esophageal cancer. From the inner to outer circles, they represent the estimates of MR-RAPS, weighted mode, weighted median, MR-Egger, and inverse-variance weighted methods, respectively. And the shades of color reflect the magnitude of the value of *p*.

**Figure 3:**
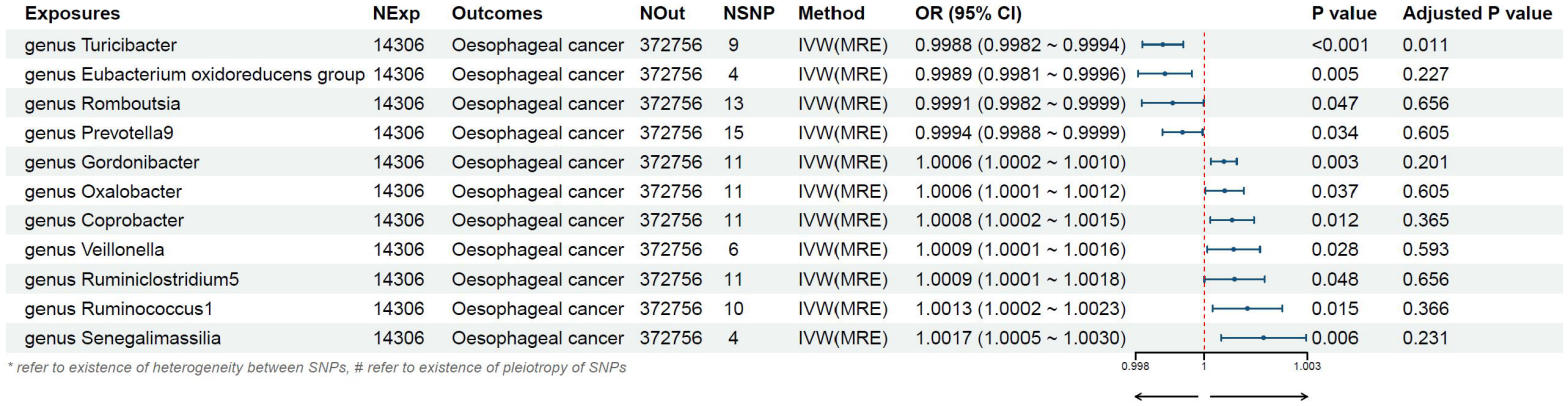
Mendelian randomization results of causal effects between gut microbiome and esophageal cancer risk.

**Figure 4:**
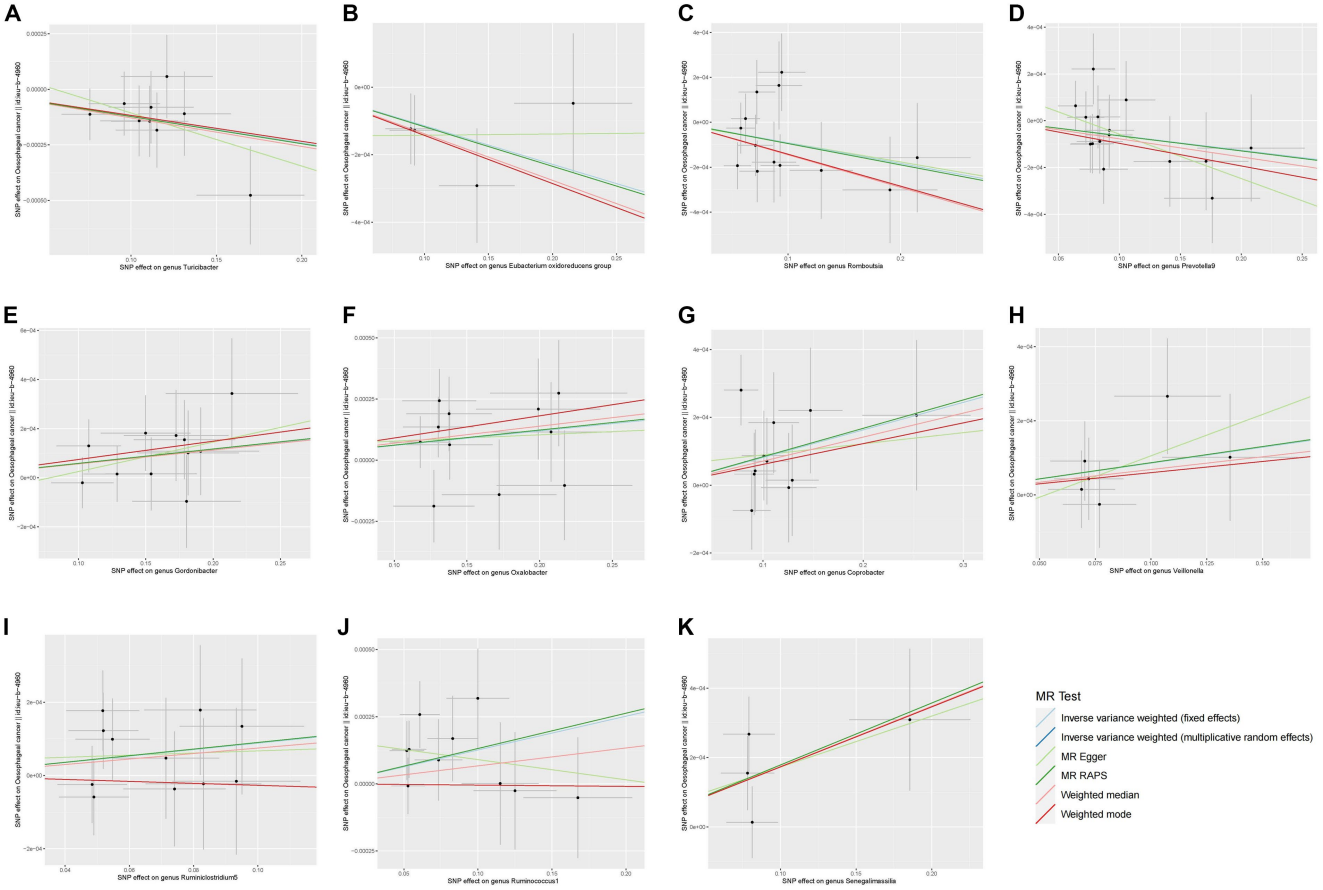
Scatter plots of the MR analyses for the association of 11 gut bacterial taxa and the risk of esophageal cancer. SNP, Single nucleotide polymorphisms; MR, Mendelian randomization.

The preliminary findings in Figure 2 demonstrate the correlation between genetically proxied gut microbiota species and the susceptibility to esophageal cancer. In addition, we have compiled all the favorable MR findings in Figure 3, displaying the odds ratios (ORs) per standard deviation (SD) increment for each exposure factor. In the event of the lack of horizontal pleiotropy, we utilized the primary analytical approach of the multiplicative random-effects IVW model. The remaining MR analysis methods were employed as sensitivity analyses to evaluate the strength of the aforementioned model. Figure 4 illustrates the causal connections between the incidence of esophageal cancer and 11 gut microbiota taxa, as uncovered by the IVW model with multiplicative random-effects.

We found that higher abundance of *Turicibacter genus* [odds ratio (OR) [95% confidence interval (CI)]: 0.9988 [0.9982–0.9994], adjusted *p* < 0.001], *Eubacterium oxidoreducens group* (OR [95%CI]: 0.9989 [0.9981–0.9996], adjusted *p*: 0.005), *Romboutsia genus* (OR [95%CI]: 0.9991 [0.9982–0.9999], adjusted *p*: 0.047), and *Prevotella9 genus* (OR [95%CI]: 0.9994 [0.9988–0.9999], adjusted *p*: 0.034) in the human gut were associated with a decreased risk of esophageal cancer (Table 1). Conversely, higher abundance of *Gordonibacter genus* (OR [95%CI]: 1.0006 [1.0002–1.0010], adjusted *p*: 0.003), *Oxalobacter genus* (OR [95%CI]: 1.0006 [1.0001–1.0012], adjusted *p*: 0.037), *Coprobacter genus* (OR [95%CI]: 1.0008 [1.0002–1.0015], adjusted *p*: 0.012), *Veillonella genus* (OR [95%CI]: 1.0009 [1.0001–1.0016], adjusted *p*: 0.028), *Ruminiclostridium5 genus* (OR [95%CI]: 1.0009 [1.0001–1.0018], adjusted *p*: 0.048), *Ruminococcus1 genus* (OR [95%CI]: 1.0013 [1.0002–1.0023], adjusted *p*: 0.015), and *Senegalimasilia genus* (OR [95%CI]: 1.0017 [1.0005–1.0030], adjusted *p*: 0.006) were associated with an increased risk of esophageal cancer (Table 1).

**Table 1:** Description of MR result.

Exposure	nSNP	OR	95%CI	IVW-derived *p* value	Power%
Genus *Turicibacter*	9	0.999	0.9982–0.9994	<0.001	5
Genus *Eubacteriumoxidoreducens* group	4	0.999	0.9981–0.9996	0.005	5
Genus *Romboutsia*	13	0.999	0.9982–0.9999	0.047	5
Genus *Prevotella9*	15	0.999	0.9988–0.9999	0.034	5
Genus *Gordonibacter*	11	1.001	1.0002–1.0010	0.003	5
Genus *Oxalobacter*	11	1.001	1.0001–1.0012	0.037	5
Genus *Coprobacter*	11	1.001	1.0002–1.0015	0.012	5
Genus *Veillonella*	6	1.001	1.0001–1.0016	0.028	5
Genus *Ruminiclostridium5*	11	1.001	1.0001–1.0018	0.048	5
Genus *Ruminococcus1*	10	1.001	1.0002–1.0023	0.015	5
Genus *Senegalimassilia*	4	1.002	1.0005–1.0030	0.006	5

No evidence of horizontal pleiotropy was found in the multi-effect analysis, as indicated by the intercept term of MR-Egger regression (all *p* > 0.05). No significant heterogeneity was detected in the heterogeneity test for the SNPs included in each MR analysis, with all *p* exceeding 0.05. All MR analysis methods are presented in Table 2, including causal estimates and sensitivity analyses. In conclusion, we utilized the MR-steiger model to verify the causal direction and confirm the overall causal effect direction of the MR analysis, and the findings indicated the accuracy of the causal direction in each MR analysis. In summary, the results of our MR analysis are generally strong and dependable. The strength of the correlation between the prevalence of 11 gut microbiota and esophageal cancer was only 0.05%.

**Table 2:** Sensitivity analysis for significant and nominal significant estimates.

Batch	Exposures	Outcomes	*Q* from IVW	*p* val_Q from IVW	*Q* from MR-Egger	*p* val_Q from MR-Egger	I^2^ from MR-Egger	*p* val of pleiotropy	Direction from MR Steiger
148	*Turicibacter (genus)*	Esophageal cancer	3.121	0.927	2.85	0.899	0.955	0.618	TRUE
76	*Eubacterium oxidoreducens group (genus)*	Esophageal cancer	1.609	0.657	1.041	0.594	0.966	0.53	TRUE
86	*Gordonibacter (genus)*	Esophageal cancer	4.222	0.937	3.97	0.913	0.931	0.628	TRUE
56	*Coprobacter (genus)*	Esophageal cancer	7.686	0.659	7.517	0.583	0.922	0.69	TRUE
136	*Ruminococcus1 (genus)*	Esophageal cancer	6.972	0.64	5.057	0.751	0.963	0.204	TRUE
142	*Senegalimassilia (genus)*	Esophageal cancer	3.01	0.39	2.992	0.224	0.986	0.921	TRUE

## Discussion

We confirmed a causal association between the abundance of 11 gut microbiota species and the risk of esophageal cancer. Specifically, an increase in the abundance of four gut microbial species was causally related to a decreased risk of esophageal cancer, while an increase in the abundance of seven gut microbial species was causally associated with an increased risk of esophageal cancer. These findings highlight the significant role of gut microbiota in the pathogenesis of esophageal cancer and provide valuable insights for further research on early diagnosis and prevention of esophageal cancer.

Previous studies have demonstrated the impact of gut microbiota on esophageal cancer. However, there is still some controversy regarding the alterations in gut microbial abundance in patients with esophageal cancer.

Li et al. (2020) found differences and similarities in the esophageal microbiota among different pathological features of esophageal squamous cell carcinoma. Among the top 10 genera with the highest relative abundance, *Veillonella* was included. Liu et al. (2019) also detected higher levels of *Veillonella* (*p* = 0.0002) in the adjacent normal tissues of esophageal squamous cell carcinoma and found that *Prevotella* and *Veillonella* genera were predominant in upper gastrointestinal tract cancer. These two genera were also found in participants with esophageal adenocarcinoma precursor lesions, such as esophagitis or Barrett’s esophagus (Liu et al., 2013).

Regarding the abundance of *Prevotella* genus bacteria and its relationship with esophageal cancer, Lv et al. (2019b) concluded that *Prevotella* genus had a higher relative abundance in esophageal squamous cell carcinoma, while the diversity of *Veillonella* genus and other Gram-negative and Gram-positive taxa was decreased, which is different from our study.

Additionally, there are other studies that have reported different results from ours. For instance, in a study conducted by Deng et al. (2021) on a Chinese population, they found a significant increase in the abundance of *Romboutsia* and a significant decrease in the abundance of *Lachnospira* genera among esophageal cancer patients at the genus level. Pan et al. (2021) also indicated in an animal experiment that several key genera, including *Romboutsia* and *Turicibacter*, were significantly associated with esophageal epithelial atrophy (*p* < 0.01 or *p* < 0.05). The underlying mechanisms of this association might involve the influence of gut microbiota on exogenous biodegradation and metabolism, as well as genomic instability (Pan et al., 2021). Conversely, our results suggest that the high abundance of *Turicibacter, Romboutsia,* and *Prevotella9* genera is associated with a reduced risk of esophageal cancer. We consider that the differences in these results may be due to regional or population variations, as well as differences in sample sizes. Therefore, further research is still needed to provide additional evidence.

In a review article, Muszyński et al. (2022) highlighted the various ways in which the gut microbiota can stimulate carcinogenesis.

Firstly, the gut microbiota has a potential impact on the host’s immune response. Münch et al. (2019) demonstrated in a mouse model of Barrett’s esophagus that a high-fat diet can induce changes in the gut microbiota, leading to increased levels of pro-inflammatory cytokines and immune cells, triggering a significant inflammatory response, and subsequently promoting a pro-tumorigenic immune phenotype to accelerate tumor growth. This study demonstrated the potential impact of the gut microbiota on esophageal diseases through inflammatory mechanisms. Proaño-Vasco et al. (2021) also described how a high-fructose diet alters the overall balance of the gut microbiota and accelerates the progression of esophageal cancer through similar mechanisms.

Secondly, the gut microbiota can produce a variety of metabolites that affect the occurrence and progression of esophageal cancer (Rooks and Garrett, 2016). For example, a study based on mouse models showed that in the distal small intestine and colon, the secondary bile acid, namely, deoxycholic acid, produced by conjugated bile acids being decoupled by the gut microbiota, can promote the development of Barrett’s esophageal and esophageal adenocarcinoma by destroying DNA (Quante et al., 2012).

Subsequently, Zhou et al. (2020) observed a significant increase in lactic acid-producing bacteria in Barrett’s esophagus, gastroesophageal reflux disease, or esophageal adenocarcinoma, suggesting that these groups exert their carcinogenic effects through dysregulation of lactate metabolism. Moreover, gut microbiota may influence esophageal adenocarcinoma by activating Toll-like receptors (TLRs) and upregulating oncogenes such as COX-2 (Yang et al., 2012; Zaidi et al., 2016; Grover et al., 2021).

Although numerous studies have investigated the potential mechanisms underlying the induction of esophageal carcinogenesis by gut microbiota, there remains some controversy regarding the causal relationship and the underlying mechanisms connecting alterations in gut microbial abundance and esophageal cancer, due to limitations in research methods and various confounding factors.

Our research offers a fresh and important insight into the causal connection between the microbiome of the human digestive system and the development of esophageal cancer. Firstly, in the absence of clinical randomized trial evidence, we unravel the potential causal impact of gut microbial composition on the risk of esophageal cancer. Secondly, we identified a causal relationship between high abundances of the following seven bacterial genera, namely *Gordonibacter, Oxalobacter, Coprobacter, Veillonella, Ruminiclostridium5, Ruminococcus1,* and *Senegalimasilia*, and an increased risk of esophageal cancer. This suggests that the elevated concentrations of these seven bacterial groups could serve as potential indicators for assessing the risk of esophageal cancer. Furthermore, it indicates the necessity for timely prevention and intervention of esophageal cancer. We propose that screening for early-stage esophageal cancer patients could theoretically be feasible by assessing the abundance of these seven bacterial groups in fecal samples. Similarly, we can reasonably infer that using appropriate antibiotics to counteract these seven bacterial groups could serve as a strategy for early prevention of esophageal cancer. Furthermore, interestingly, we also found that high abundance of the genera *Turicibacter, Eubacterium oxidoreducens group, Romboutsia,* and *Prevotella9* may decrease the risk of esophageal cancer, providing valuable guidance for clinical translational applications. These four taxa could potentially serve as components of probiotic supplements for oral administration in high-risk populations for esophageal cancer prevention. However, further large-scale clinical validation is needed to explore the therapeutic potential of probiotic supplementation in esophageal cancer (Kaźmierczak-Siedlecka et al., 2020; Liu et al., 2021).

To the best of our knowledge, there is limited research on the characteristics of gut microbiota in esophageal cancer patients, and the majority of existing studies are observational. This study represents the first investigation into the causal relationship between human gut microbiota and esophageal cancer using large-scale GWAS data and Mendelian randomization analysis. Moreover, previous MR studies have predominantly focused on a limited number of microbial taxa as exposure indicators to assess their overall relationship with digestive tract tumors. In contrast, we comprehensively examined the impact of gut microbiota on esophageal cancer risk by utilizing a larger dataset comprising 150 taxa. Given the substantial variations among different phyla, classes, and orders of microbial taxa, we specifically employed data at the genus and family levels to obtain more specific conclusions. Importantly, this study encompassed a sizable sample of 372,756 Europeans, with 8,970,465 SNPs available for analysis. The study population consisted of 740 esophageal cancer patients and 372,016 control individuals, which substantially mitigated sampling errors to a certain extent.

Nonetheless, this study has certain limitations. Firstly, although our Mendelian randomization (MR) study demonstrated a causal association between gut microbiota and esophageal cancer, the underlying mechanisms remain unclear and require further investigation for elucidation. Furthermore, all the GWAS data included in this study were derived from European populations, which, while minimizing the confounding effect of population stratification, raises the need for validation of the causal relationship conclusions in other non-European populations, such as Asians. Additionally, the low statistical power of certain correlation findings in this study may increase the occurrence of type II errors. Thirdly, because summary-level data was used in this study, it was not possible to conduct stratified analysis using individual-level data. Moreover, given that the ratio estimation technique assumes a linear correlation between exposure and outcome, it is important to acknowledge the potential existence of a non-linear association between the two variables in this research. Finally, when collecting gut microbiota samples, due to the differences between the patient’s diet, medication, lifestyle, etc., it is inevitable to have an impact on the abundance of microbiota.

Despite these potential limitations, our study rigorously controlled for quality in various aspects, including the assumptions of MR analysis, data anomalies and heterogeneity, and confounding factors in causality. Through a series of sensitivity analyses, we have confirmed the reliability and robustness of our causal estimation conclusions, specifically demonstrating the strong causal association between 11 gut microbiota and the occurrence of esophageal cancer based on large-scale GWAS data. In comparison to previous observational studies, our research approaches the causal relationship between gut microbiota and esophageal cancer from a different perspective by employing genetic variation as instrumental variables to simulate the randomized controlled trial (RCT) methodology. These 11 bacterial taxa can serve as biomarkers for esophageal cancer prevention or as alternative therapeutic approaches for reducing the risk of esophageal cancer.

## Conclusion

Our study demonstrated the causal relationship between human gut microbiota and esophageal cancer through two-sample Mendelian randomization analysis. Specifically, high abundance of the genera *Gordonibacter, Oxalobacter, Coprobacter, Veillonella, Ruminiclostridium5, Ruminococcus1,* and *Senegalimasilia* was associated with an increased risk of esophageal cancer, while high abundance of the genera *Turicibacter, Eubacterium oxidoreducens group, Romboutsia,* and *Prevotella9* was associated with a decreased risk of esophageal cancer. These 11 bacterial taxa can serve as biomarkers for esophageal cancer prevention or as alternative therapeutic approaches for reducing the risk of esophageal cancer. In comparison to previous observational studies, our research approaches the causal relationship between gut microbiota and esophageal cancer from a different perspective by employing genetic variation as instrumental variables to simulate the RCT methodology. Our findings provide a theoretical basis for guiding clinical practice and further indicate directions for future research. We hope that healthcare professionals and researchers will pay more attention to the detection of gut microbiota in early screening and prevention of esophageal cancer, thereby identifying additional risk predictors and potentially beneficial microbial taxa, in order to implement primary prevention measures promptly for esophageal cancer. This represents the most significant clinical implication of our study.

## Data availability statement

Publicly available datasets were analyzed in this study. Study analyzes publicly available datasets. This data can be found here: https://mibiogen.gcc.rug.nl/ and https://gwas.mrcieu.ac.uk/.

## Ethics statement

Our analysis did not involve the collection of original data but utilized published studies or publicly available GWAS summary data; therefore, ethical approval was not required.

## Author contributions

XG: Writing – original draft. ZW: Writing – original draft. BL: Writing – review & editing. YC: Writing – review & editing.
